# Comparison of findings identified at traditional invasive autopsy and postmortem computed tomography in suicidal hangings

**DOI:** 10.1007/s00414-022-02874-2

**Published:** 2022-08-12

**Authors:** James R. Lyness, Anthony J. Collins, Jane E. Rutty, Guy N. Rutty

**Affiliations:** 1 The State Pathologist’s Department, Belfast, UK; 2 Royal Victoria Hospital, Belfast, UK; 3 The Faculty of Health and Life Sciences, De Montfort University, Leicester, UK; 4 East Midlands Forensic Pathology Unit, University of Leicester, Leicester, UK

**Keywords:** Forensic, Suicide, Hanging, Postmortem computed tomography, PMCT, Invasive autopsy, Trauma, Natural disease

## Abstract

Cases of suicidal hanging are a common death referred for medico-legal autopsy throughout the world. Although some advocate using postmortem computed tomography (PMCT) without traditional invasive autopsy (TIA) to investigate such deaths, others reject this approach. There is currently limited evidence to guide practice. In this context, the TIA reports and PMCT images of 50 cases of suspected suicidal hanging during an 11-month period were reviewed. The reviewers were blinded to the findings of the other modality. A Cohen’s Kappa coefficient (K) was calculated to assess agreement between TIA and PMCT across a range of pertinent findings. This analysis demonstrated perfect agreement for identification of a ligature (*K* = 1.00) and a strong level of agreement for identification of a ligature suspension point (*K* = 0.832) but only a minimal level of agreement for overall ligature mark (*K* = 0.223). PMCT demonstrated a weak level of agreement for fractures of hyoid bone (*K* = 0.555) and thyroid cartilage (*K* = 0.538). Three probable fractures not identified at TIA were identified on PMCT. TIA was shown to be superior in the identification of intramuscular and laryngeal fracture–related haemorrhage/bruising whereas PMCT was superior to TIA in identifying body gas deposition. There was overall good correlation between the natural disease and trauma identified elsewhere in the body during the TIA and PMCT. The study demonstrates that PMCT can assist the investigation of suspected suicidal hangings. However, the accuracy of many findings is limited, and if it is used as an alternative to the TIA, potentially pertinent findings, such as fractures of the laryngeal cartilages, could be missed.

## Introduction

In 2018, a total of 6507 deaths were registered as suicides within the UK, equating to a national average of 11.2 deaths/100,000. The group of hanging, strangulation and suffocation remained the most commonly chosen methods, accounting for 59.4% and 45.0% of suicides amongst males and females, respectively [1]. Whilst this group was not differentiated further, in our experience, we would expect hanging to account for the vast majority of these cases. In the UK, all suspected suicides would normally require an investigation by a coroner. This would typically include a postmortem examination and for the majority of cases a traditional invasive autopsy (TIA). However, in recent years, the use of less invasive techniques, such as postmortem-computed tomography (PMCT), in death investigation has been increasing throughout many regions of the UK [2]. As suicidal hangings constitute a common referral to coroners, it is extremely important that those involved in death investigation have an evidence-based understanding of the information that can be obtained from a PMCT scan compared to the TIA in such cases.

A review of the literature identified multiple manuscripts documenting specific findings on PMCT in relation to hangings and general papers outlining local approaches to death investigation and/or the overall application of PMCT in a wide range of causes of death [3–35]. There was consensus that PMCT was superior at detecting gas deposits in various anatomical scenarios specific to hanging, including the so-called gas bubble sign related to laryngeal cartilage fractures [5], soft tissue emphysema and pneumomediastinum [7–9, 23]. However, these findings were not present in all cases. In addition, there was general agreement that PMCT was less able to detect soft tissue haemorrhage in comparison to TIA [3, 9, 19, 23], the detection of which is important as it not only indicates antemortem injury, but if excessive may also suggest additional neck trauma prior to a ligature being applied, e.g. in a masked homicide. The majority of manuscripts also concluded that PMCT could detect laryngeal fractures, but there was considerable variation in the reported detection rates [3, 8–10, 20, 21, 23, 35]. Furthermore, many of the studies failed to set a reference standard or to complete the PMCT and/or TIA blind to the findings of the other modality. Indeed, none of the previous studies specifically assessed the agreement between PMCT and TIA in hangings and therefore critically assess the application of PMCT. There were also no large-scale studies.

During both TIA and PMCT, practitioners document information, e.g. trauma and natural disease, which may assist the coroner in investigating the circumstances leading to death. In cases of hanging, practitioners should also describe the ligature and ligature mark, as well as additional injuries to the skin and internal structures of the neck. In this context, our study aimed to assess whether PMCT can provide similar information, in these important areas, to the coronial investigation in cases of suspected suicidal hanging compared to TIA. The results should provide an evidence base for coroners, pathologists and others working within death investigation to justify the extent of the postmortem examination for this common cause of death.

## Materials and methods

### Subject recruitment

All suspected suicidal hangings that had undergone PMCT and TIA within the State Pathologist’s Department (SPD), Belfast, between 1st March 2020 and 31st January 2021 were considered for the study. Permission to use the case material for research was granted by the Coroner for Northern Ireland. Cases were excluded if the relevant anatomical regions, including the head, neck, chest, abdomen and pelvis, were not examined in full during TIA and PMCT. In addition, cases were excluded if the size of the deceased was greater than the CT-scanners 70 cm bore diameter.

#### PMCT

PMCT scanning was completed prior to the commencement of the TIA using a mortuary located 32-slice scanner (Somaton GO.UP Siemens Healthcare, Germany) which utilised automatic dose modulation. The body was retained within a sealed body bag and was placed on the CT scanner in as close to a supine position as rigor mortis, if present, allowed. Images of the head, neck, chest, abdomen and pelvis were acquired in every case. No gantry tilt was available. The scans and subsequent image reconstructions, including soft tissue and bone algorithms, were completed using pre-set protocols (Table 1). The CT images were reviewed by a single consultant radiologist with 24 years of clinical radiology experience and Fellowship of the Royal College of Radiologists, UK. The radiologist had no access to the TIA findings or report and was simply aware that each case was a suspected suicidal hanging. Images were viewed using an OsiriX Lite 12.0.1 DICOM viewer on a 2.5 GHz Intel Core i5 iMac computer attached to a RadiForce RX340 EIZO high-definition medical monitor which featured precise calibration compliant with the DICOM Part 14 standard.

**Table 1:** Study PMCT protocols

Scan region	KV	Quality ref. mAs	Slice thickness (mm)	Increment (mm)	Window	Kernel	Pitch	Rotation (s)
Head	130	191	5.00	5.00	Base orbita Inner ear	HR40 HR60	0.55	1
	130	191	1.50	1.00	Base orbita Inner ear	HR40 HR60	0.55	1
Neck	130	129	2.00	2.00	Spine disc Bone	BR40 BR60	0.80	1
	130	129	1.50	1.00	Spine disc Bone	BR40 BR60	0.80	1
Chest, abdomen and pelvis	130	150	5.00	5.00	Abdomen Bone	BR40 BR60	0.80	0.8
	130	150	1.50	1.00	Abdomen Bone	BR40 BR60	0.80	0.8

### Invasive autopsy

Following the PMCT, a TIA was completed by one of four consultant forensic pathologists with ≥ 18 years of autopsy experience and Fellowship of the Royal College of Pathologists, UK (RCPath). The autopsy entailed external and internal examinations, including the cranial, chest and abdominal cavities. An anterior neck dissection was completed in all cases. The examinations were to the standards set by the RCPath [36]. The pathologists did not review the PMCT images prior to the TIA. Furthermore, the pathologist did not have access to the radiologist’s report prior to the completion of the autopsy report.

### PMCT and TIA analysis

Once fully completed, the reports from the TIA and PMCT examinations were reviewed. The positive and negative findings, including those pertinent to hanging and any additional evidence of trauma/injury, signs of natural disease and identifying features, were collated and entered into a spreadsheet in IBM SPSS statistics software version 26. A Cohen’s Kappa coefficient was calculated to test the agreement between the data generated by PMCT and TIA. It was considered important that any PMCT findings were accurate if they could be used in a coronial or police investigation. The thresholds for each level of agreement were therefore set to levels previously used in the clinical setting as published by McHugh [37].

## Results

One hundred and twenty-five suspected suicidal hangings were referred to SPD during the 11-month study period. Of these, 63 underwent PMCT and TIA. The decision to undertake PMCT was primarily based on the consultant forensic pathologist and daily service pressures within the mortuary. There were no exclusions made on the basis of the size of the deceased and the restrictions of the CT-scanner bore diameter. However, following review of the PMCT images, 13 cases were excluded as the neck structures were not entirely captured. A total of 50 cases were therefore included in the study.

### Case demographics

The study sample included 38 males (76%) and 12 females (24%). The average age of the decedents was 37.1 years (range 17–70; *SD* 13.75). The largest number of cases was within the 20–29 age bracket (*n* = 14) and case numbers decreased slightly through the following three decades. The postmortem interval was calculated for each case, based on the difference between the date and time when the individual was found dead and the date and time of the PMCT and TIA. The average postmortem interval was 31 h (range 7–87; *SD* 15.2).

### Findings specific to hanging

In 11 (22%) of the 50 cases, the decedent was fully suspended when found hanging, i.e. no part of the body was in contact with another surface, causing the entirety of the body weight to be held by the ligature around the neck. In 28 cases (56%), part of the body remained in contact with another surface, e.g. the floor/ground or an object used to gain height. In the remaining 11 cases (22%), it was not known if the weight of the body was fully or partially suspended.

The TIA external examination revealed conjunctival petechial haemorrhages in 29 cases (58%). Six of these were fully suspended decedents, 17 partially suspended and 6 in those where the degree of suspension was not known. This corresponded to 60.7%, 54.5% and 54.5% of each group, respectively. Perhaps not unexpectantly petechial haemorrhages were not identified during PMCT.

### Ligature

In 20 cases (40%), the ligature used in the hanging was submitted with the deceased and remained around the neck of the decedent at the time of PMCT and TIA. PMCT identified all 20 of the ligatures present and did not suggest the presence of any ligature in the remaining 30 cases. This perfect agreement produced a Cohen’s Kappa coefficient (K) of 1.00.

**Fig. 1:**
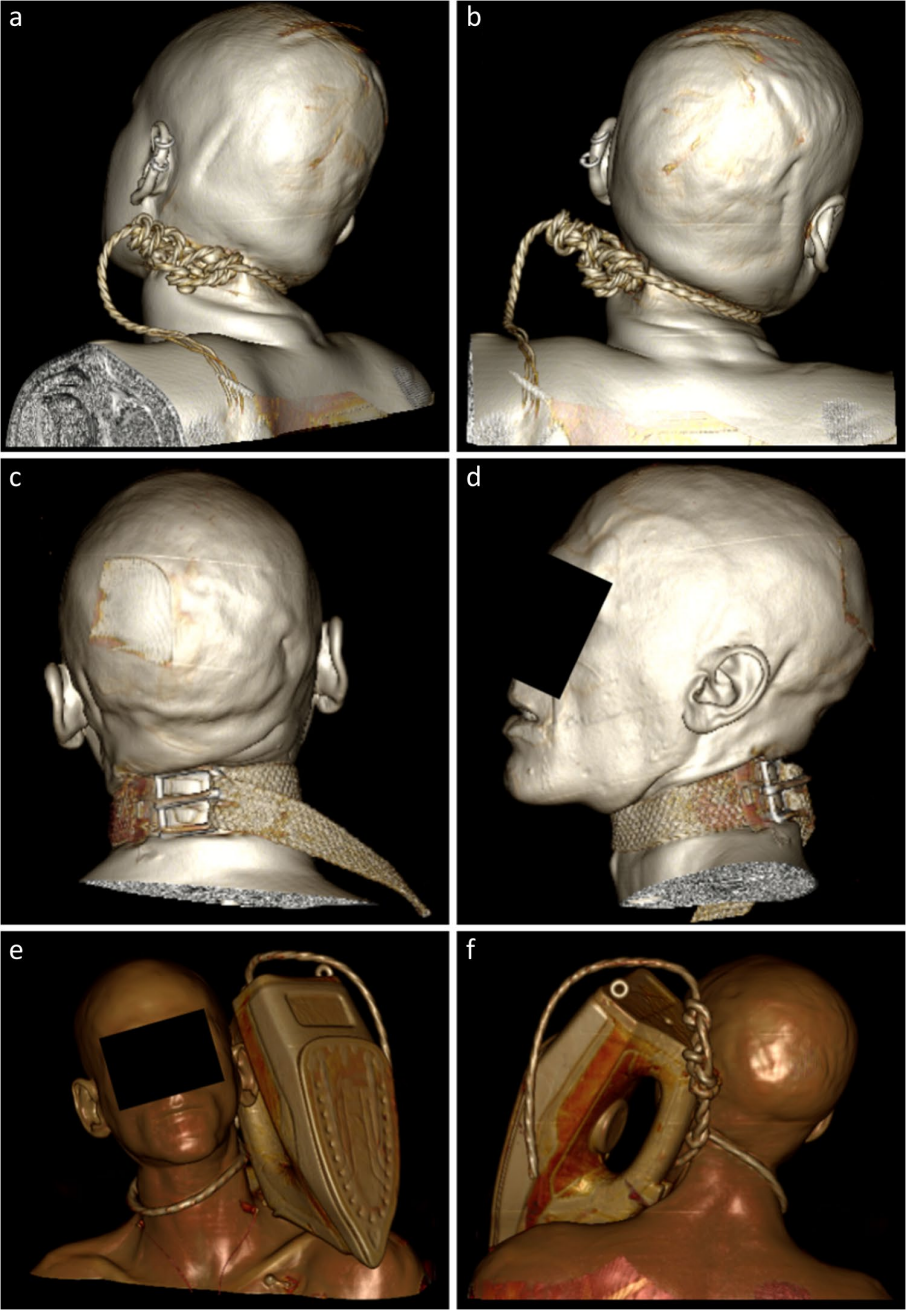
3D-volume-rendering images demonstrating range of ligatures in study cases. Posterior views (**A**, **B**) of complex knot within a rope in case 16. Posterior (**C**) and lateral (**D**) views of a woven clothing belt in case 9. Anterior (**E**) and posterior (**F**) views of a clothes iron in case 46

**Fig. 2:**
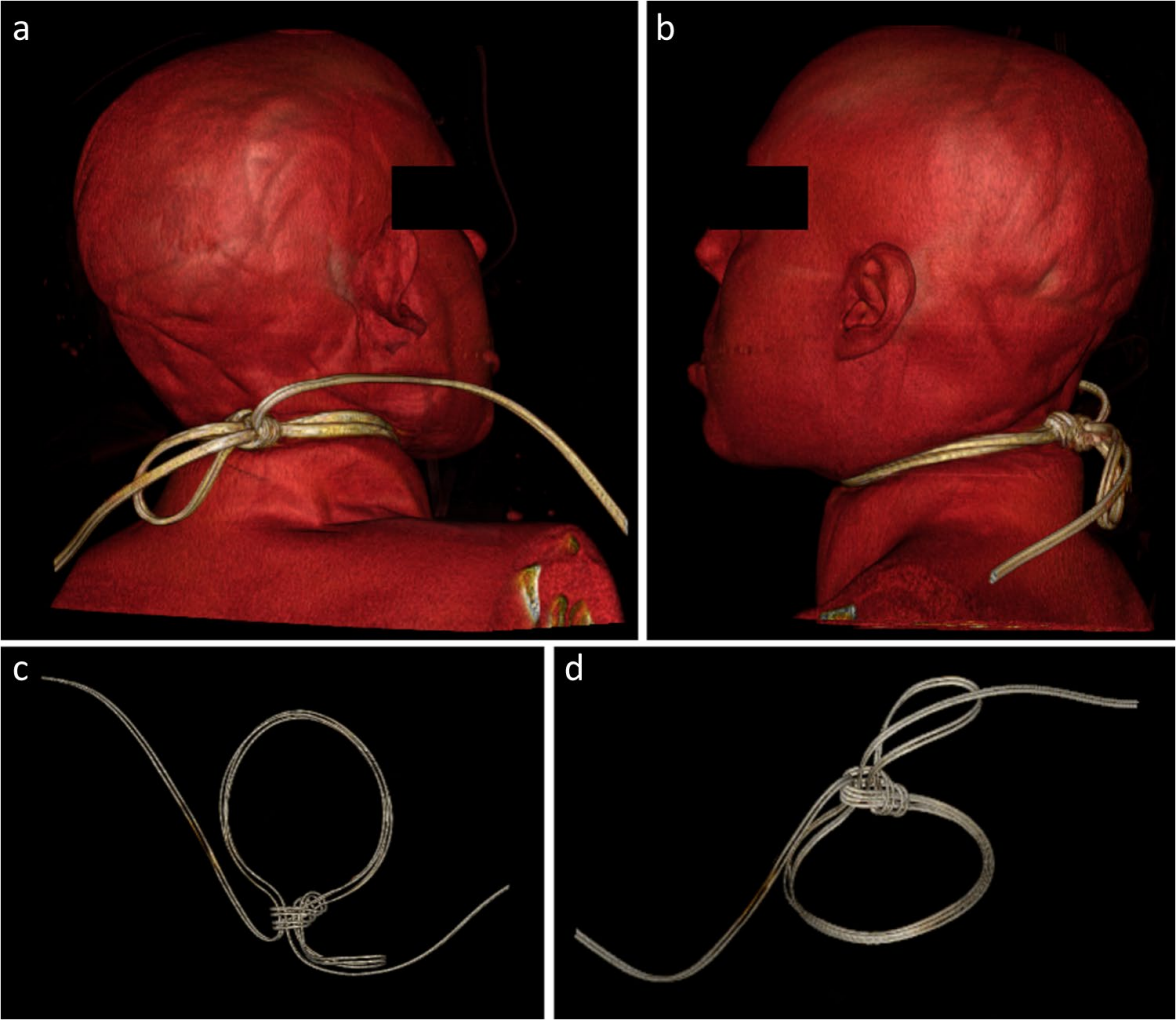
3D-volume-rendering images of wire used as ligature in case 14. Posterior views (**A**, **B**) and ligature only views (**C**, **D**)

**Fig. 3:**
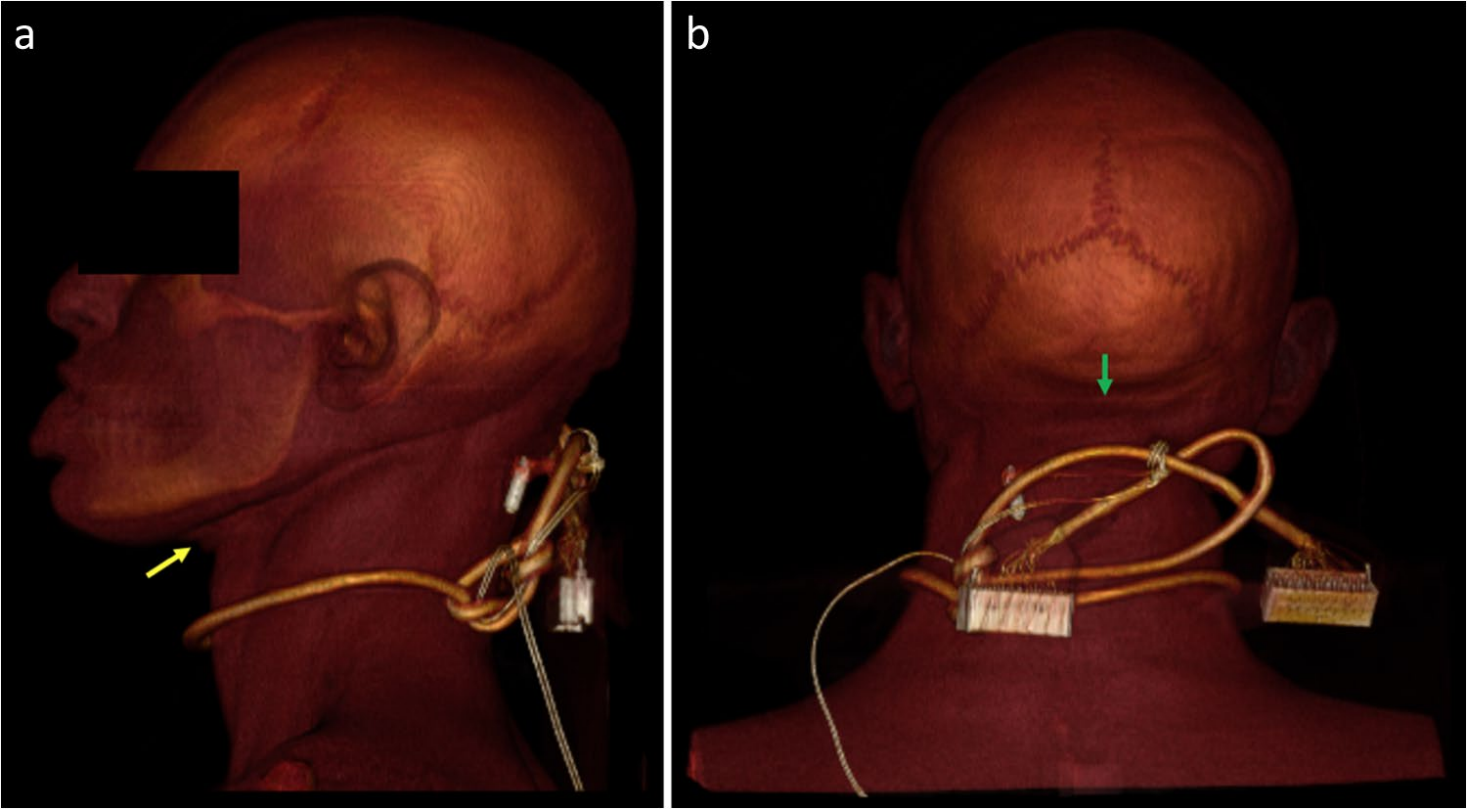
3D-volume-rendering images (**A**, **B**) demonstrating movement of the ligature, a scart lead, from ligature mark (yellow arrow) and suspension point (green arrow) in case 42

In addition, it was noted that the PMCT images frequently provided a detailed archive of the ligature. Of those identified, it was almost always possible to specify the ligature type, such as a rope, belt and wire cable, using a combination of standard axial images and 3D-volume-rendering techniques (Figs. 1). Furthermore, the PMCT images could allow knot detail to be reviewed and with windowing techniques, the head and/or neck could be removed from the generated image(s) (Fig. 2). The latter of these provides sanitised images, potentially useful for court purposes. Of note, however, the ligature did not always remain within or in close proximity to the ligature mark (Fig. 3) and therefore careful review of the DICOM images is recommended.

### Ligature mark and suspension point

A ligature mark was identified in 49 cases (98%) during TIA. In the single case in which no ligature mark was observed, a soft woollen scarf was used as the ligature and there was partial suspension of the body with the decedent found in a kneeling position. PMCT failed to identify the ligature mark in an additional 6 cases, therefore 14% (*n* = 7) of the overall study population. Using Cohen’s Kappa test, a result of 0.223 was achieved demonstrating a minimal level of agreement between the modalities.

**Fig. 4:**
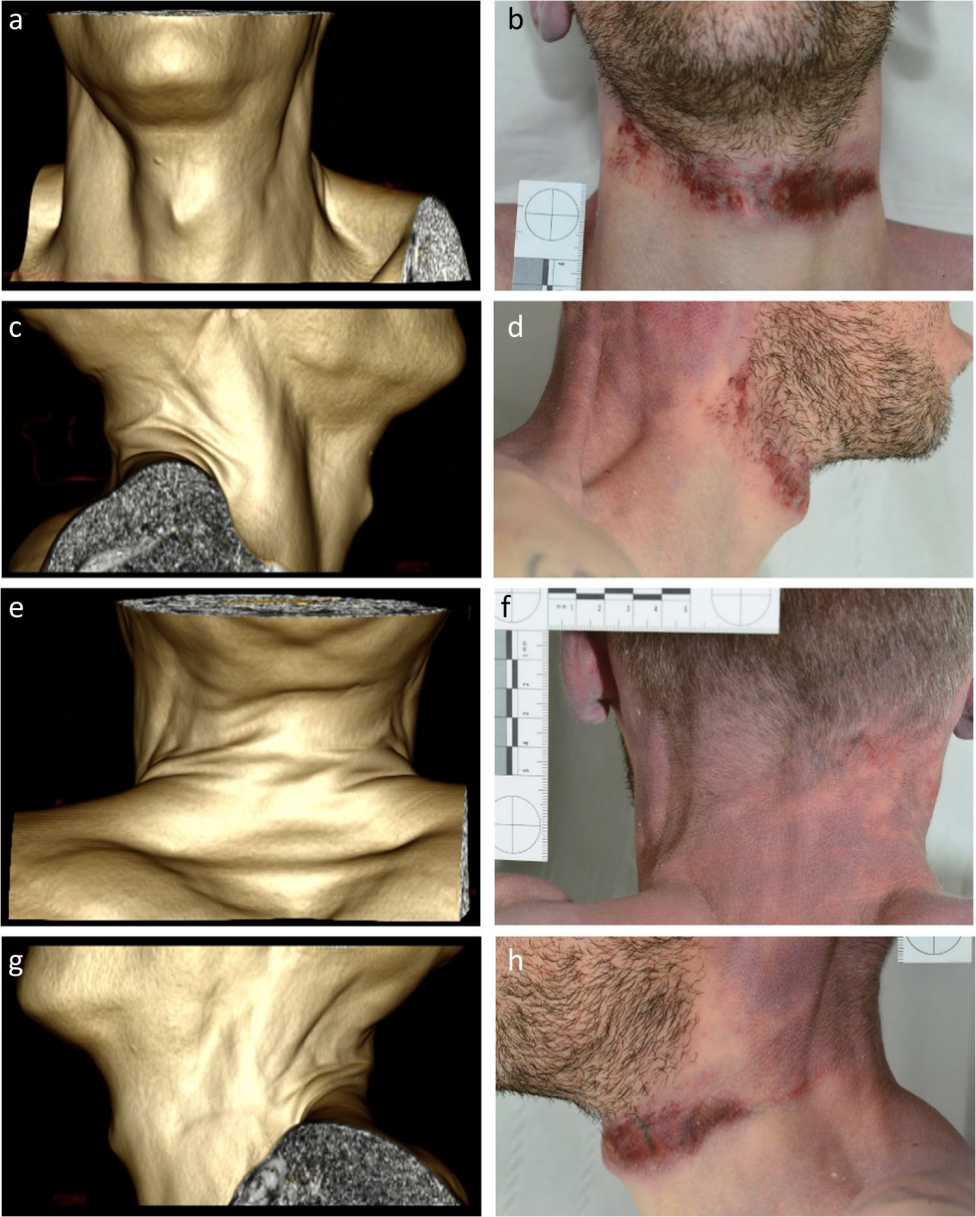
3D-volume-rendering images and macroscopic photographs of neck, front (**A**, **B**), right (**C**, **D**), posterior (**E**, **F**) and left (**G**, **H**), demonstrating difficulties with identifying a shallow ligature mark on PMCT

In each of the 7 cases where no ligature mark was identified during PMCT, the ligature mark described at TIA had no depth to it, comprising bands of pale/pink skin (Fig. 4). In addition, the cases involved a relatively soft ligature in 4 cases and partial suspension in 5 cases.

**Fig. 5:**
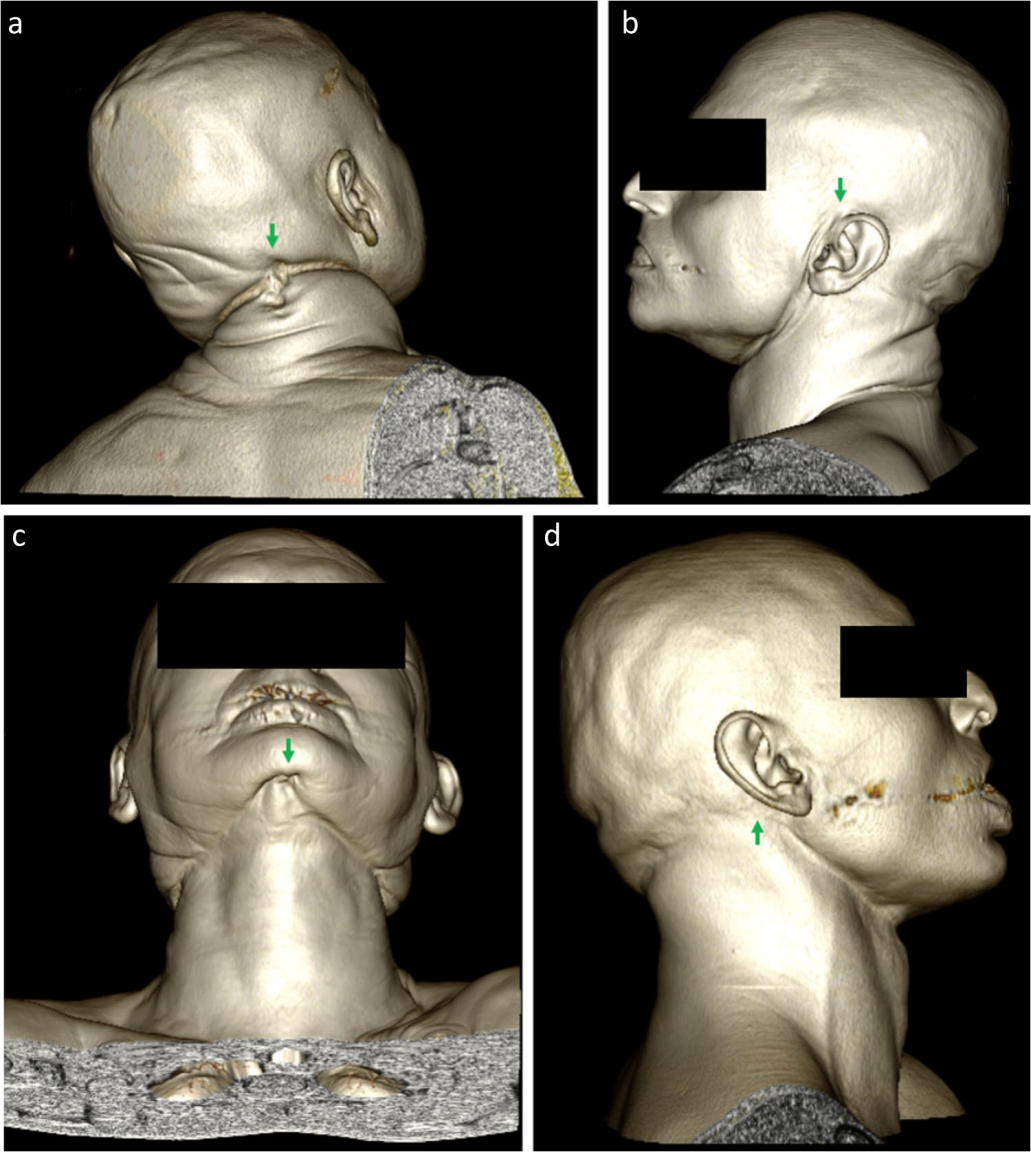
3D-volume-rendering images demonstrating variation in the position of suspension points identified during PMCT (green arrows). Posterior with knot indentation in case 21 (**A**), in proximity of the left ear in case 11 (**B**), anterior/submental in case 39 (**C**) and in proximity of the right ear in case 18 (**D**)

The ligature suspension point was identified in 48 cases (96%) during TIA. PMCT did not identify the suspension point in the same two cases as TIA, as well as a further 5 cases. This corresponded to the same 7 (14%) cases where the ligature mark was not identified on PMCT. Of the 43 suspension points identified on PMCT (Fig. 5), there was complete concordance with the position recorded at TIA. A Cohen’s Kappa coefficient was calculated as 0.832, consistent with a strong level of agreement between the modalities.

In addition to the above, we noted that during review of the PMCT scans, the identification of the ligature mark and point of suspension was more difficult if there were prominent skin folds within the neck region. This was observed in cases where the deceased was obese and/or if the neck was in an obscure position. Furthermore, we found that windowing techniques were useful to confirm the position of the suspension point, as identified during TIA, in individuals with a large volume of scalp hair.

### Intramuscular haemorrhage

Bruising of the anterior neck muscles was identified in only a minority of cases, 3 (6%), during TIA. In all of these cases, the haemorrhage/bruising was reported as slight. PMCT did not specifically identify intramuscular haemorrhage, but soft tissue swelling and vascular congestion were recorded in 12 cases (24%), potentially representative of congestion secondary to hanging and/or hypostasis. Two of these cases also had TIA-identified intramuscular haemorrhage, but a Cohen’s Kappa coefficient was calculated as 0.189 demonstrating no significant agreement.

### Laryngeal fractures

**Fig. 6:**
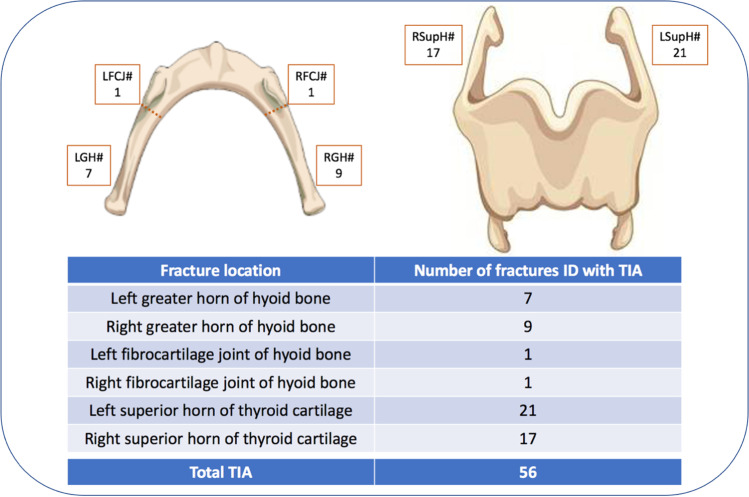
Frequency of fractures identified by anatomical location with TIA

**Fig. 7:**
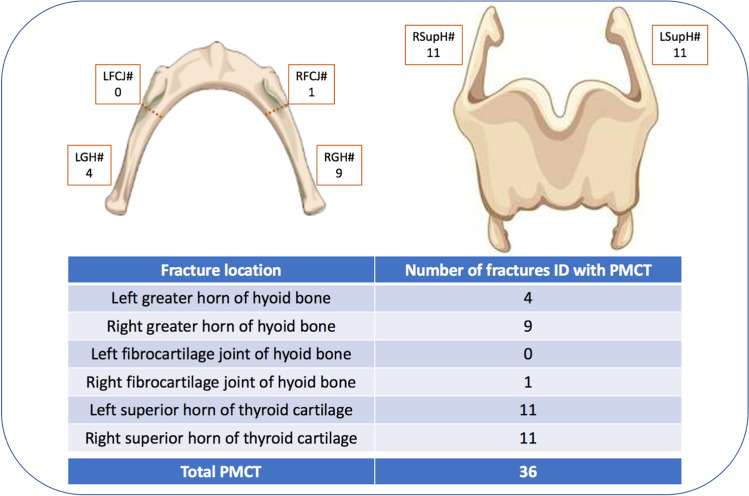
Frequency of fractures identified by anatomical location with PMCT

A total of 56 fractures were identified during TIA, within 26 cases (52%). Review of the PMCT scans revealed 36 fractures within 22 cases (44%). Fractures of the superior horns of the thyroid cartilage were the most frequently identified in both modalities (Figs. 6 and 7). Furthermore, TIA recorded that 1 of the superior horns of the thyroid cartilage was fractured in 2 places in 3 cases. However, whilst PMCT identified one of these ‘double fractures’, the injuries were counted as a single fracture within the fracture statistics.

No fractures of the cricoid cartilage were recorded during TIA or PMCT. However, in a single case, the radiologist noted low attenuation.

**Fig. 8:**
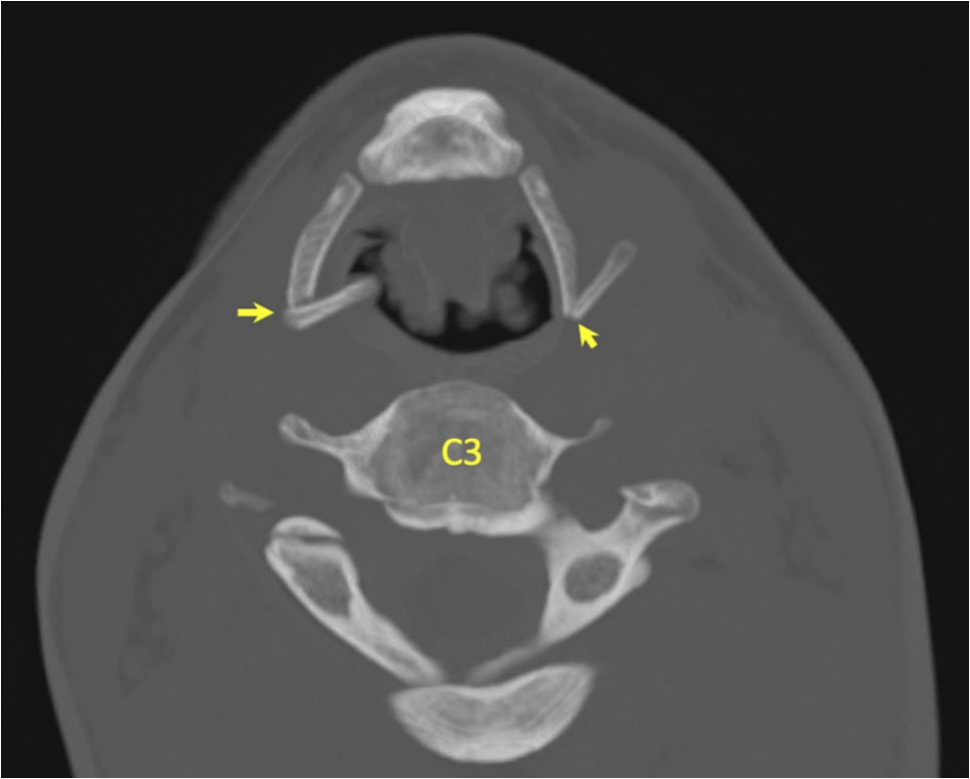
3D-MPR axial image demonstrating bilateral hyoid bone fractures, identified during TIA and PMCT in case 12

**Fig. 9:**
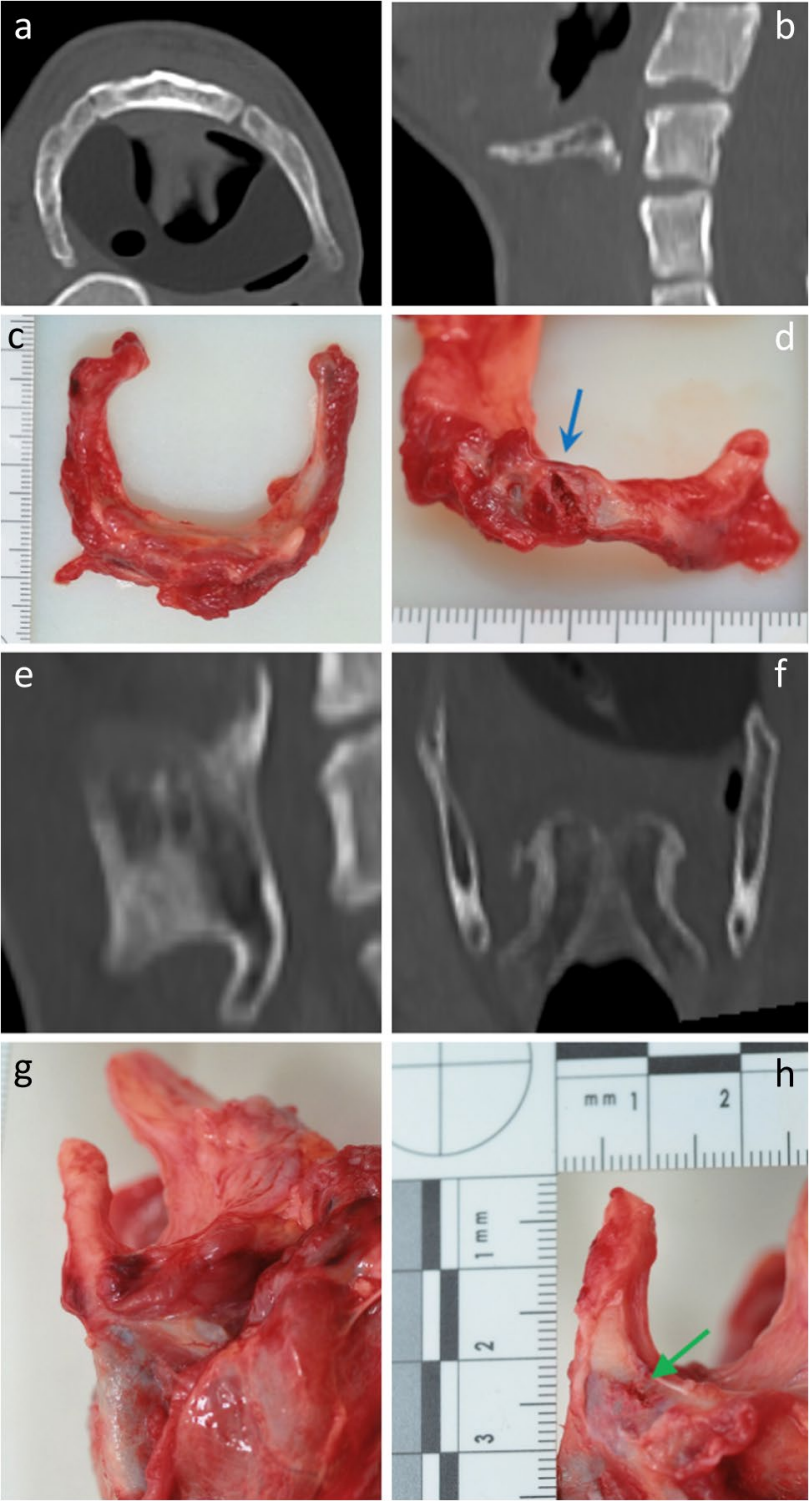
3D-MPR axial (**A**) and sagittal (**B**) images of the hyoid bone and sagittal (**E**) and coronal (**F**) images of the thyroid cartilage with no definite fracture identified on PMCT. Macroscopic photographs of corresponding fractures in hyoid bone (**C**, **D**) and thyroid cartilage (**G**, **H**)

Of the 56 laryngeal fractures identified during TIA, 30 (53.57%) were also identified on PMCT (Fig. 8). Of the 26 (46.43%) fractures not identified on PMCT, 8 were within the hyoid bone and 18 were within the thyroid cartilage, representing 44.44% and 47.37% of each group, respectively (Fig. 9).

A fracture or fractures were identified within the hyoid bone in 13 cases (26%) during TIA, corresponding to 18 individual fractures, 8 left-sided and 10 right-sided. Hyoid fracture(s) were identified in 13 cases with PMCT, totalling 14 individual fractures, 4 left-sided and 10 right-sided. However, despite the similar case/fracture numbers, crosstabulation revealed that 8 of the 18 fractures recorded during TIA were not identified on PMCT. Conversely, 4 fractures were reported on PMCT, but not during TIA. Cohen’s Kappa coefficients were calculated for left- and right-sided fractures, as well as overall for the hyoid bone (Table 2). The overall result of 0.555 demonstrates a weak level of agreement. Furthermore, in comparison to TIA, PMCT demonstrated a sensitivity of 55.6% and a specificity of 95.1% for hyoid bone fractures.

**Table 2:** Summary of statistical analyses for hyoid bone fractures

Hyoid bone fracture	Cohen’s Kappa (K)	Asymptotic standard error	Sensitivity (%)	Specificity (%)
Left-sided	0.440	0.185	37.5	97.6
Right-sided	0.625	0.139	70.0	92.5
Overall	0.555	0.113	55.6	95.1

Fracture(s) of the thyroid cartilage were identified in 22 cases (44%) during TIA, including 38 individual fractures, 21 left-sided and 17 right-sided. Thyroid cartilage fracture(s) were recorded in 18 cases (36%) with PMCT, corresponding to 22 individual fractures, 11 on each side. Crosstabulation revealed that 18 of the 38 fractures recorded during TIA were not identified on PMCT. Conversely, 2 fractures were reported on PMCT, but not during TIA. Cohen’s Kappa coefficients were again calculated for left- and right-sided fractures, as well as overall for the thyroid cartilage (Table 3). The overall result of 0.538 demonstrates a weak level of agreement with a PMCT sensitivity of 52.6% and specificity of 96.8% compared to TIA.

**Table 3:** Summary of statistical analyses for thyroid cartilage fractures

Thyroid cartilage fracture	Cohen’s Kappa (K)	Asymptotic standard error	Sensitivity (%)	Specificity (%)
Left-sided	0.473	0.118	47.6	96.6
Right-sided	0.610	0.120	58.8	97.0
Overall	0.538	0.085	52.6	96.8

**Fig. 10:**
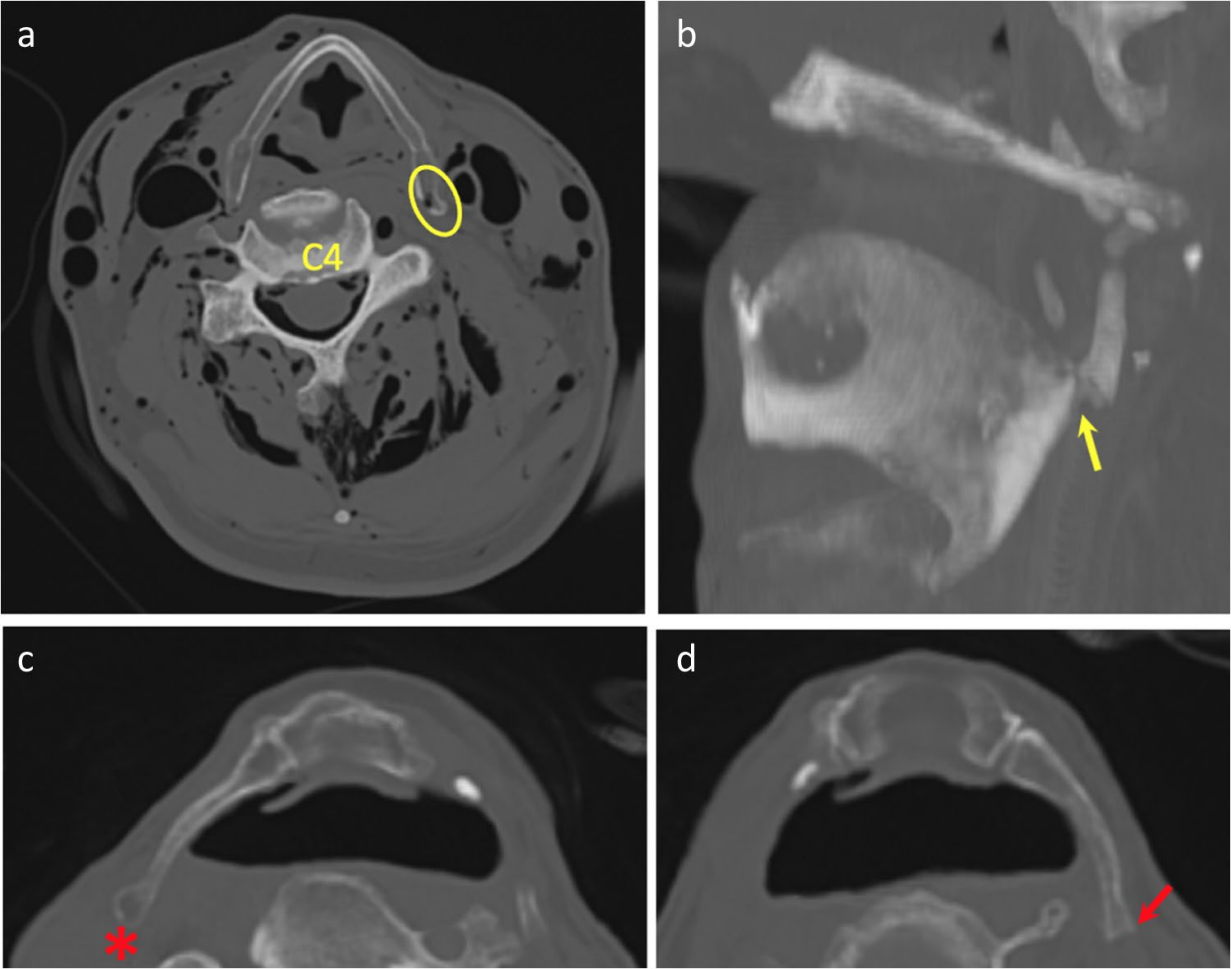
Axial (**A**) and 3D-MPR (**B**) generated images of fracture of left superior horn of thyroid cartilage identified with PMCT only (yellow arrow). A fracture-associated gas-bubble (oval) was also identified, but this case had extensive decomposition gas artefact. Axial images (**C**, **D**) of squared tip of left greater horn of hyoid bone misinterpreted as fracture (red arrow) in contrast to rounded tip of right greater horn (*)

As discussed above, PMCT identified 6 fractures not described during TIA, 4 within the greater horns of the hyoid bone and 2 within the superior horns of the thyroid cartilage. Following review of the images, one of these represented a definite missed diagnosis during TIA (Fig. 10B). Two were considered probable missed fractures during TIA. However, three of the fractures described only on PMCT probably represented anatomical variations misdiagnosed as fractures on PMCT (Fig. 10D).

Bruising/haemorrhage associated with the laryngeal fractures was identified during TIA in 19 (38%) cases. These included 6 of the 13 cases with fractures of the hyoid bone and 13 of the 22 cases with fractures of the thyroid cartilage. No fracture-associated bruising/haemorrhage was specifically mentioned by the radiologist reporting the PMCT scans. However, soft tissue swelling and asymmetry were reported in some cases, including those without fractures.

### Laryngeal anomalies

**Fig. 11:**
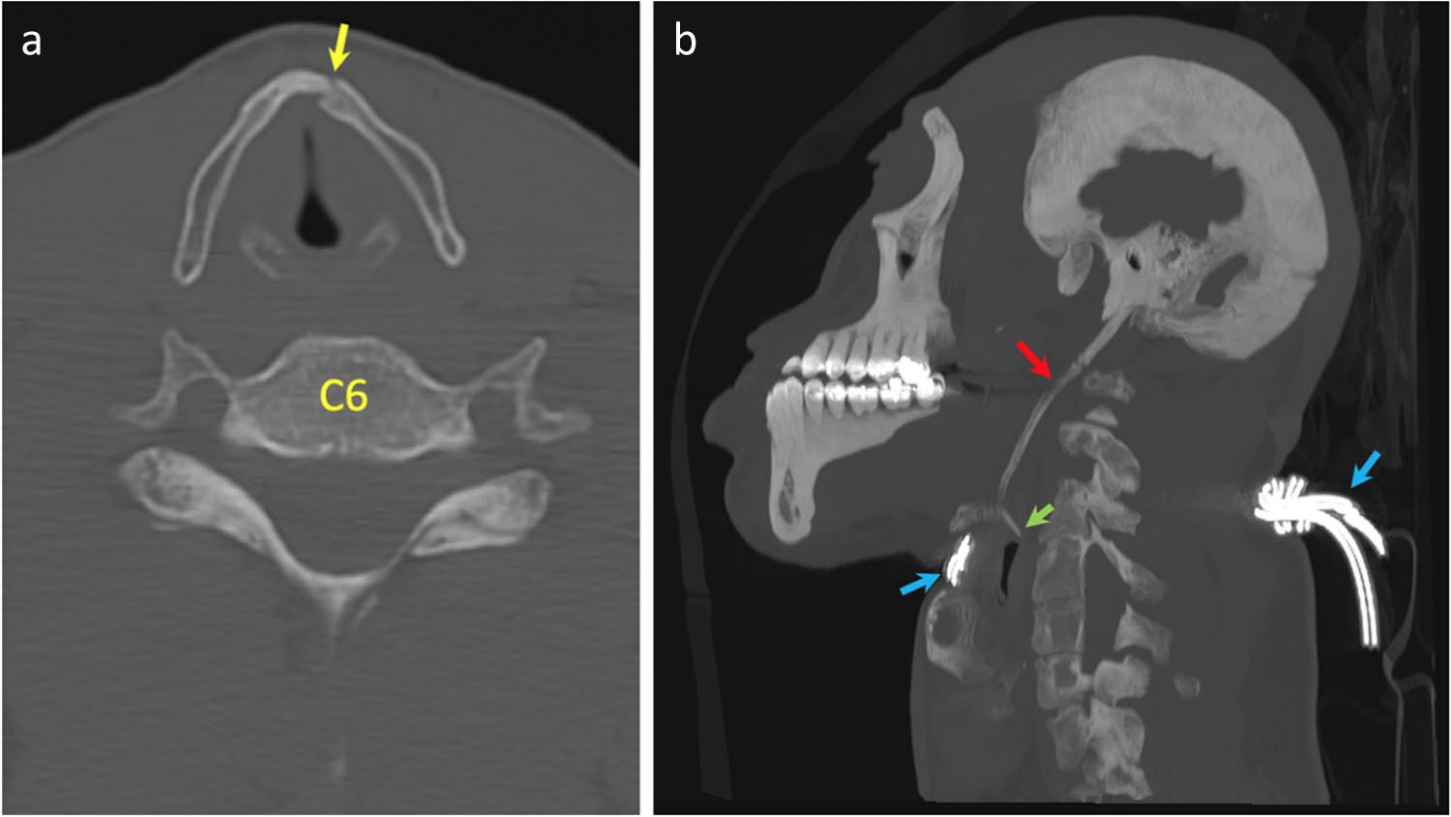
Axial image (**A**) demonstrating defect of left side of laryngeal prominence (yellow arrow) in case 13. 3D-MPR generated image (**B**) of calcified stylo-hyoid ligament (red arrow) in case 14 with fracture of right greater horn (green arrow) and wire ligature (blue arrows) also demonstrated

Anomalies within the hyoid bone were identified in 3 cases (6%) during TIA. These included 2 sets of calcified stylo-hyoid ligaments and a single misshapen right greater horn, possibly representative of a previous fracture. PMCT identified 10 anomalies within 9 hyoid bones, including non-fusion (*n* = 3), widening of the fibrocartilage joint (*n* = 4), osteophytes (*n* = 2) and calcified stylo-hyoid ligaments (*n* = 1) (Fig. 11b).

The commonest anatomical variation in the thyroid cartilage was the presence of a triticeal cartilage(s) within the lateral thyrohyoid ligament. These were identified within 24 cases (48%) during TIA, 4 (8%) solitary left-sided, 5 (10%) solitary right-sided and 15 (30%) bilateral. Therefore, a total of 39 triticeal cartilages were described during TIA, but only 3, equating to 7.69%, were also identified with PMCT, 2 left-sided and 1 right-sided. However, PMCT identified 4 additional anatomical variations/deviations within the thyroid cartilage, all of which were due to asymmetrical deformity (Fig. 11a).

### Gas deposition

**Fig. 12:**
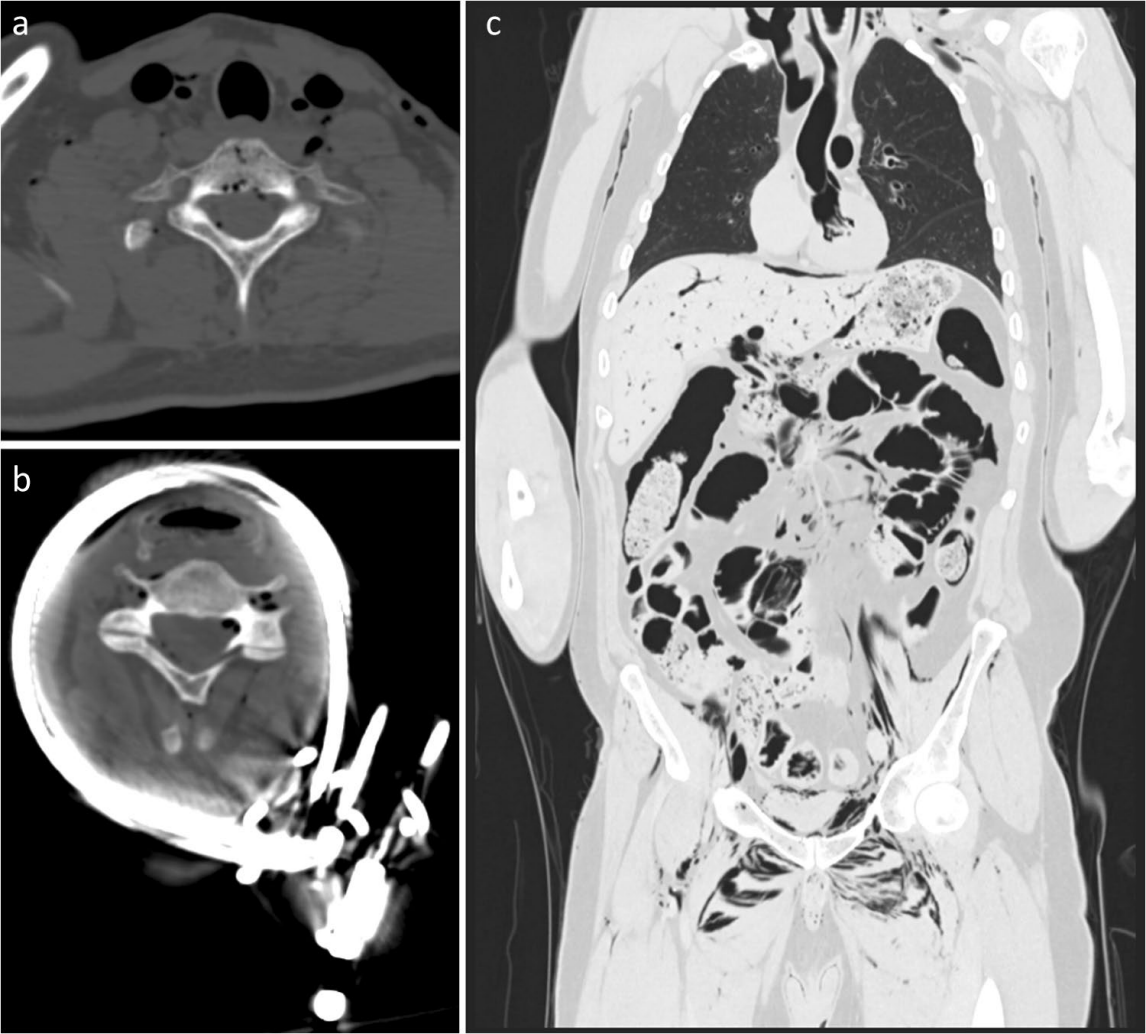
3D-MPR axial images of severe gas artefact within the neck at the level of C7 (**A**) and C4 with plastic coated metal cable ligature (**B**). Coronal 3D-MPR image demonstrating gas artefact throughout remainder of body (**C**). All case 29

Gas deposits were identified within the neck structures in 23 cases (46%) on PMCT. These had an average postmortem interval of 34.52 h (SD 16.5), whereas those without subcutaneous gas had an average postmortem interval of 28.00 h (SD 13.67). Five of the 23 cases had extensive subcutaneous emphysema within the neck structures, and all of these had concomitant decomposition gas artefact throughout the remainder of the body, including the mediastinum (Fig. 12). The average postmortem interval for these cases was 37.80 h (*SD* 15.09). The remaining 18 cases in which gas deposits were identified had only minimal focal deposits within the neck structures, including within blood vessels (*n* = 5), peri-clavicular soft tissues (*n* = 5), anterior neck muscles (*n* = 6) and peri-laryngeal soft tissues (*n* = 3).

**Fig. 13:**
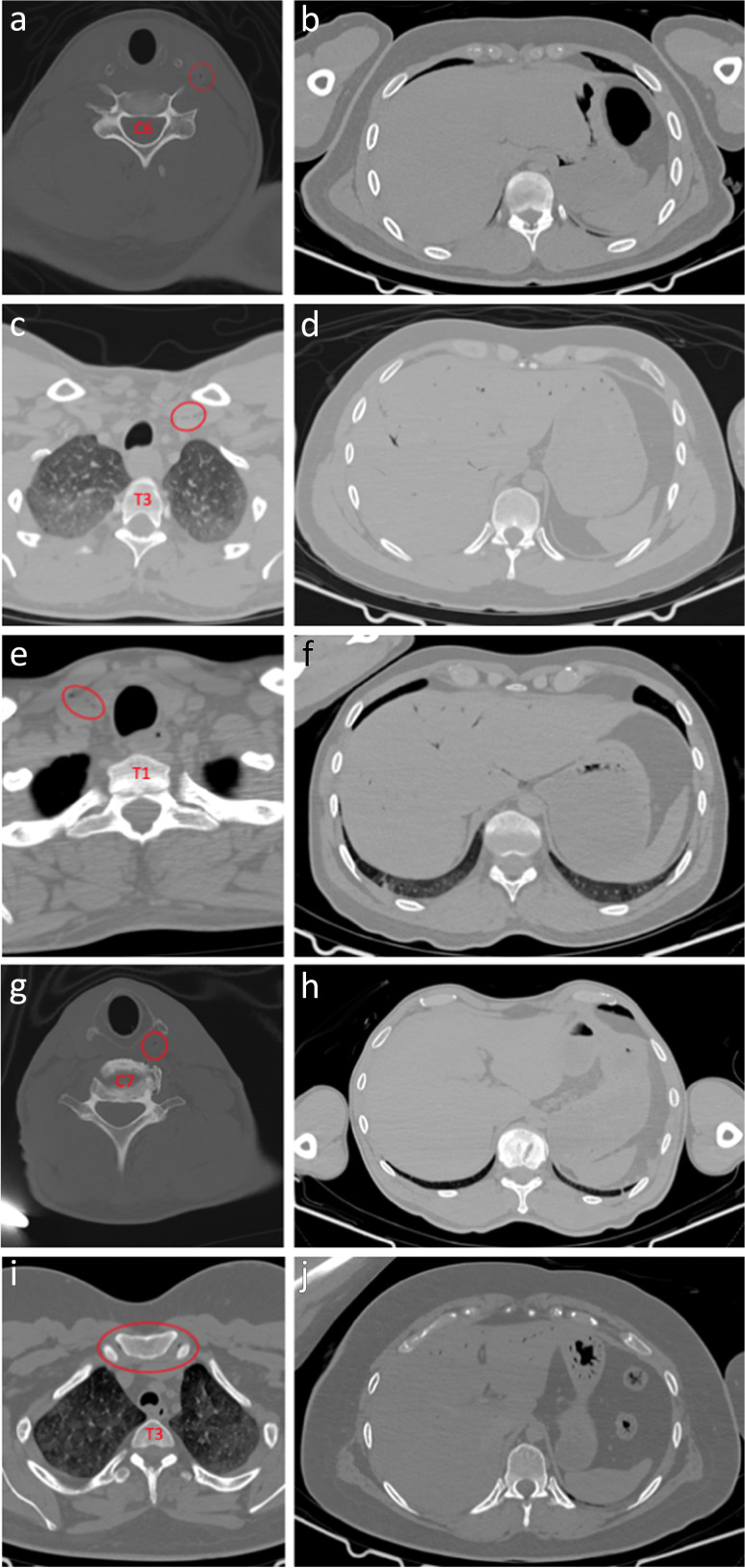
3D-MPR axial images demonstrating gas deposits in left common carotid artery in case 16 (**A**), in left internal jugular vein in case 27 (**C**), anterior to right internal jugular vein in case 28 (**D**), in left-sided peri-laryngeal tissues in case 33 (**G**) and in manubrio-clavicular joints in case 31 (**I**). Hepatic images for each case (**B**, **D**, **F**, **H** and **J**)

Gas artefact was identified within the liver in 23 cases (46%) during PMCT. Of these, the average postmortem interval was 32.57 h (*SD* 17.27), compared to an average postmortem interval of 29.04 h (*SD* 13.32) in cases where no hepatic gas was identified. Crosstabulation revealed that of the 23 cases where hepatic gas was detected, 18 also had gas deposits within the neck structures. Therefore, only 5 cases where gas deposits were identified within the neck did not have concomitant gas within the liver (Fig. 13).

**Fig. 14:**
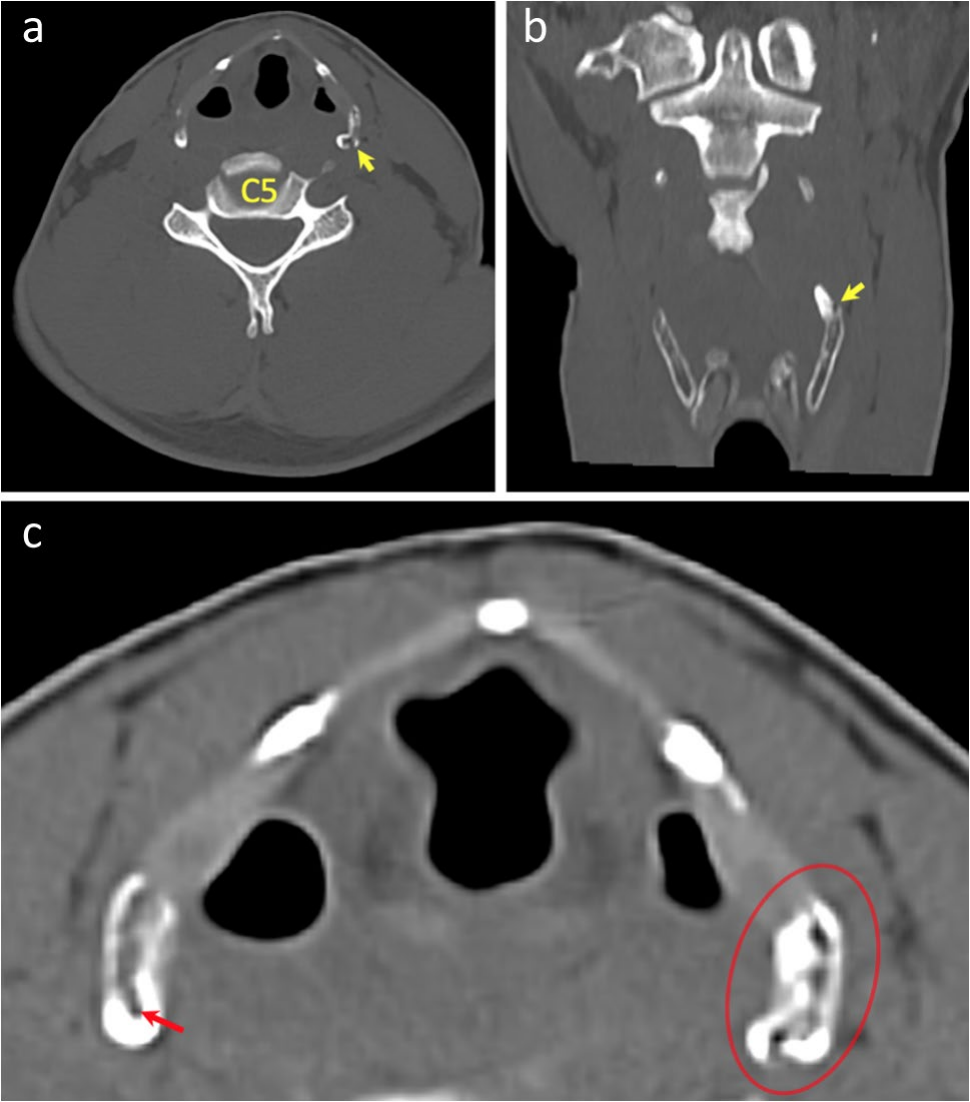
3D-MPR generated axial (**A**, **C**) and coronal (**B**) images demonstrating a medially displaced fracture of the left superior horn of the thyroid cartilage (yellow arrows) in case 11. Gas bubbles were also identified within the fracture (red oval) and possibly within the non-fractured right superior horn (red arrow)

**Fig. 15:**
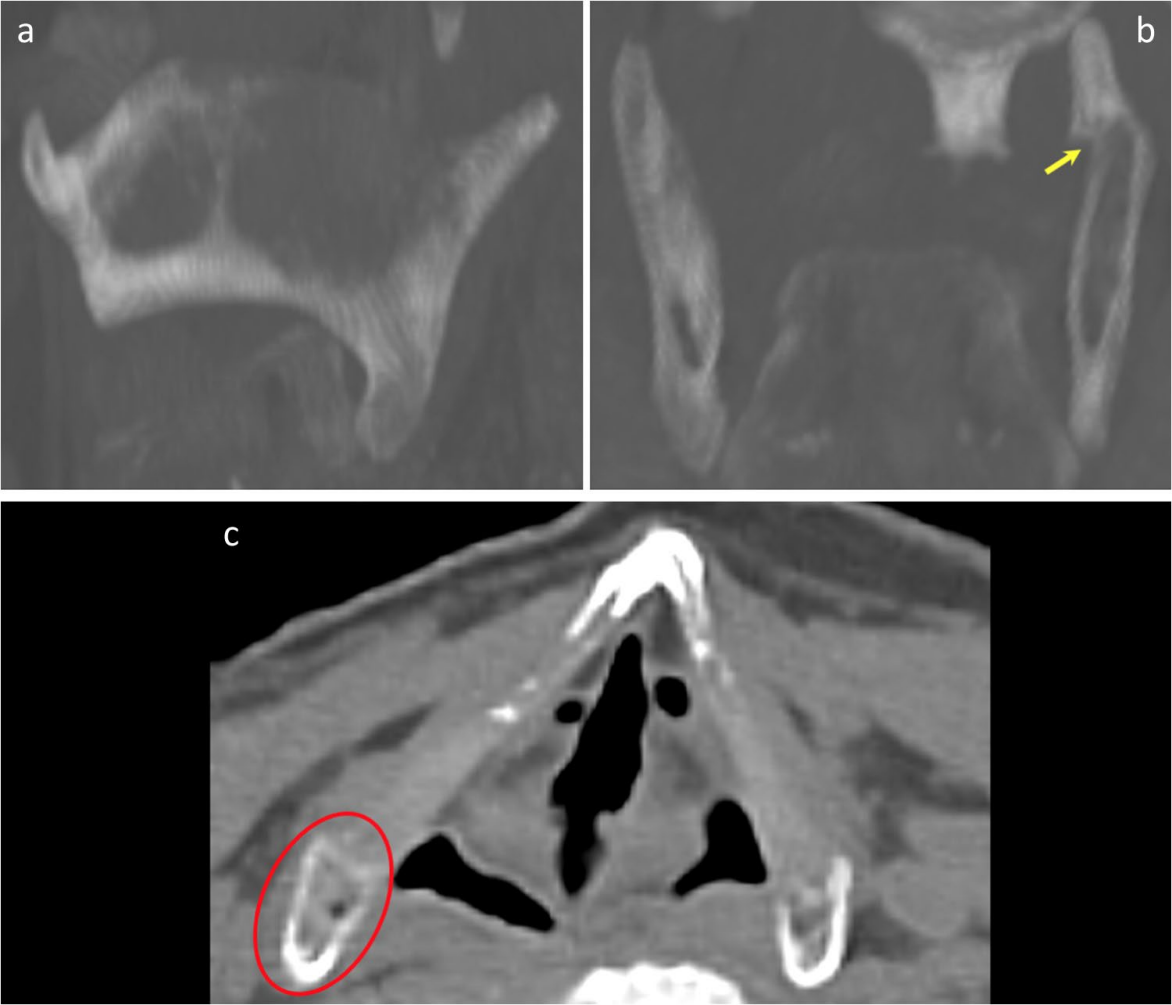
3D-MPR generated images demonstrating right lateral view of thyroid cartilage (**A**), a displaced fracture of the left superior horn of the thyroid cartilage (**B**) and a gas bubble within the right superior horn (**C**) in case 23. TIA revealed bilateral fractures of the superior horns

Gas bubbles were identified in proximity of fractures reported during TIA and/or PMCT in 4 (8%) cases. All of these were within the superior horns of the thyroid cartilage (Table 4). In only one of these cases, case 11, was the gas bubble associated with a fracture identified on both modalities. However, this case also contained a tiny gas bubble within the right superior horn which was not reported as fractured on either TIA or PMCT (Fig. 14). In addition, two of the gas bubbles identified were associated with fractures considered missed during TIA, cases 7 and 24, but the latter of these had extensive decomposition gas artefact (Fig. 10A, B). In the final case, case 23, the gas bubble identified was associated with the right superior horn, but there was no displacement of the cartilage and the radiologist did not record a definite fracture. In contrast, the displaced fracture identified on PMCT in this case did not have any associated gas bubble (Fig. 15). TIA revealed bilateral fractures of the superior horns in this case.

**Table 4:** Summary of identified gas bubbles and associated fractures

Case No	Air bubble location	Fracture reported onTIA	Fracture reported on PMCT	Hepaticgas
7	Right superior horn	No	Yes	Minimal
11	Left superior horn	Yes	Yes	None
	Right superior horn	No	No	
23	Right superior horn	Yes	No	None
24	Left superior horn	No	Yes	Extensive

### Cervical spine

There were no fractures of the cervical spine identified within any of the 50 cases. However, due to the often suboptimal position of the neck, primarily due to rigor mortis, the radiologist could not confidently confirm or exclude ligamentous injury, e.g. based on the relationship between vertebrae. In 4 cases (8%), osteoarthritic changes were identified within the cervical spine during PMCT.

### Natural disease

Due to the relatively young age of the majority of the decedents included in the study, average 37.1 years, the vast majority had only minor/incidental natural disease identified during TIA and/or PMCT. It was not a primary objective of this study to offer a detailed scientific analysis of specific conditions. However, in general, there was good correlation between the natural disease identified during TIA and PMCT. Whilst a number of discrepancies between the natural disease detected during TIA and PMCT were identified (Table 5), there were no missed diagnoses critical to providing the medical cause of death. Indeed, all of the discrepancies, as summarised in Table 5, could be classified as category 3 errors, i.e. likely to be of little clinical significance, in accordance with definitions previously recommended by RCPath [38]. Furthermore, the discrepancies largely represent the limitations of each modality, e.g. in the absence of angiography, it was inevitable that minor coronary artery disease would not be detected on PMCT. Similarly, the dissection required to diagnose sinusitis during TIA could never be justified in a case of suspected suicidal hanging.

**Table 5:** Summary of natural diseases identified by a single modality

Additional finding with TIA	No. of cases	Additional finding with PMCT	No. of cases
Coronary artery atheroma (mild/moderate)	11	Sinus disease	6
Cardiomegaly/left ventricular hypertrophy	2	Prominent nasal turbinate	2
Bicuspid aortic valve	1	Diverticular disease	1
Slight emphysema	5	Enlarged lymph nodes	3
Slight cerebral atrophy	1	Bilateral inguinal hernias	1
Old cerebrovascular accident	1	Spinal osteoarthritis	4
Hepatosteatosis	1	Possible lung granulomata	1
Thyroid gland atrophy	2	Thymic remnant	1
Meckel’s diverticulum	1	Prominent appendix	1
Renal scarring	1	Thickened urinary bladder wall	2
Early pleural plaques	1	Posterior lung tree bud	1
Pyonephrosis	1		
Benign prostatic hypertrophy	1		

### Trauma

Additional internal trauma was also documented with each modality. The most common finding recorded during both TIA and PMCT was sternal and anterior rib fractures, detected in 9 cases (18%). These were attributed to resuscitative chest compressions. A number of relatively small bruises of the undersurface of the scalp were identified during TIA, but none were recorded with PMCT. In addition, old cortical contusions, secondary to previous head injury, were identified in 2 cases (4%) during TIA. These were not seen during initial reporting of the PMCT scan, nor following review of the scans after completion of the study. A single old wedge compression fracture of T12 was identified on PMCT and not diagnosed during TIA. However, similar to the variation in natural disease detected, none of this additional trauma was critical to the cause of death or circumstances of the case and again could be classified as category 3 errors.

### Toxicology

Potential drug and alcohol toxicity forms another part of the investigation of a suspected suicidal hanging by the coroner. Blood samples were retained in all 50 cases in this study. A urine sample was also available in 45 cases (90%). All of these samples were submitted for alcohol analysis with average concentrations of 77.24 mg/100 ml (maximum 268 mg/100 ml; *SD* 93.53) in the blood and 104.58 mg/100 ml (maximum 335 mg/100 ml; *SD* 117.96) in the urine. Of note, however, 25 cases (50%) had no alcohol in the bloodstream and 18 had none in the urine. Of the 21 cases (42%) over the current legal limit for driving in Northern Ireland, 80 mg/100 ml, the average blood alcohol was 176.48 mg/100 ml.

Fourteen cases (28%) also had toxicology/drug analysis completed. No drugs were detected in one of these cases. The drugs recorded in the remaining cases comprised a range of common pharmaceutical medications and drugs of abuse.

**Fig. 16:**
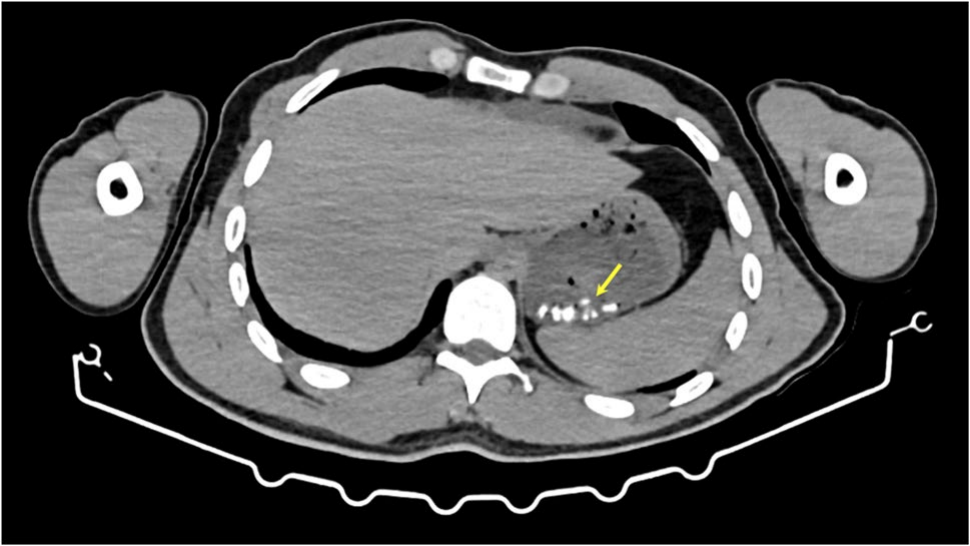
Axial image demonstrating multiple high attenuation fragments within the stomach (yellow arrow) in case 18

Whilst alcohol and/or drug analyses can be ordered during a TIA or a minimally invasive PMCT, autopsy findings may assist practitioners to make this decision. In particular, high attenuation fragments, suggestive of medication tablets, were identified within the stomach in 6 cases (12%) on PMCT (Fig. 16). In four of these, tablet collections were also found during TIA.

### Foreign bodies

Finally, PMCT identified a couple of foreign objects not recorded during TIA. These included a subcutaneous contraceptive device within the right arm and metallic foreign bodies in proximity of the right maxillary sinus. However, there was no inferences drawn from these additional findings and both were classified as category 3 discrepancies.

## Discussion

Our study has shown a varied set of results for the agreement obtained between PMCT and TIA in cases of suspected suicidal hanging. There was perfect agreement (*K* = 1.00) between the modalities for the identification of the ligature, a conclusion that would be supported by other manuscripts [4, 9, 18]. We have also shown that PMCT provides a detailed archive, allowing future examination of the ligature characteristics and knot detail, and sanitised images, potentially useful for court purposes. However, despite 43 of the 50 ligature marks being correctly identified with PMCT, statistical analysis only demonstrated a minimal level of agreement with TIA (*K* = 0.223). In contrast, our study demonstrated a strong level of agreement (*K* = 0.832) between PMCT and TIA in the identification of a suspension point. We believe that we are the first to provide statistical data in this area. Furthermore, we found that the identification of the ligature mark and suspension point during PMCT was more difficult if there were prominent skin folds within the neck region. This was observed in cases where the deceased was obese and/or if the neck was in an obscure position, typically secondary to rigor mortis.

Internally, we found that PMCT could not identify intra-muscular haemorrhage, but such injuries were only present in a minority of the study cases. On the other hand, PMCT did demonstrate soft tissue swelling and vascular congestion within the neck, but analysis showed no significant agreement between the modalities (*K* = 0.189). Perhaps more importantly, our study has shown only a weak level of agreement between PMCT and TIA in fractures of the hyoid bone (*K* = 0.555) and thyroid cartilage (*K* = 0.538). This would be similar to the results of Hueck et al., who reported a Cohen’s Kappa coefficient of 0.54 for injuries of the laryngeal cartilages [9], albeit the group subsequently used different thresholds for the stated level of agreement. We also experienced the same difficulties in diagnosing laryngeal fractures as outlined by other groups, including decreased ossification [4, 19, 21], the size of the lesions [21] and non-displaced fractures [21]. However, there is considerable variation in the rate of detection of laryngeal fractures using PMCT within the medical literature. Deininger-Czermak et al. used a high-resolution PMCT scan of the larynx in addition to a whole body PMCT and reported more radiologically identified fractures than during TIA [8]. Furthermore, Decker et al. [35] concluded that PMCT was equal to TIA in the detection of laryngeal fractures and Le Blanc-Louvry et al. [20] found almost perfect concordance between PMCT and TIA with fractures of the hyoid bone. However, these conclusions were based on small numbers of fractures, 12 and 5, respectively. In support of radiological assessment, our study recorded a small number of fractures identified on PMCT only, which would suggest that TIA is not an infallible technique.

It is well recognised that PMCT is superior to TIA in the identification of gas deposition within the body [9]. In hanging cases, fracture-associated gas bubbles and subcutaneous emphysema have been described, but again there is some variation in results/opinions across manuscripts [3, 5, 7–9, 12, 17, 19, 23]. Schulze et al. reported that a ‘gas bubble sign’ was highly suggestive of laryngeal fractures in hanging cases with a positive-predictive-value of 95% and overall accuracy of 83%, but the study excluded decomposed decedents [5]. In contrast, the two papers by Deininger-Czermak et al. identified fracture-related gas bubbles in only 20% [8] and 16.67% of cases [19], and additional gas bubbles in proximity of the laryngeal structures without any fractures in 16% of cases [8]. Our study recorded gas bubbles in proximity of fractures in only 8% of cases, and only one of these fractures was identified during both TIA and PMCT. We also identified gas bubbles in proximity of non-fractured laryngeal structures. In addition, our study identified gas deposits within the neck structures during PMCT in 23 cases (46%), 18 of which also had gas deposition within the liver. Indeed, analysis showed that these cases had a higher average postmortem interval compared to those where no subcutaneous gas was observed, 34.52 and 28.00 h, respectively. Furthermore, in all 5 cases where subcutaneous emphysema within the neck was extensive, there was decomposition gas artefact throughout the rest of the body and the average postmortem interval was even longer (37.80 h). In the remaining 18 cases in which gas deposits were identified, these were described as minimal focal deposits within the neck structures. Therefore, overall, we did not find gas deposition a particularly useful indicator of hanging and any deposits should be interpreted in relation to decomposition of the body.

In addition, our study demonstrated overall good correlation in the natural disease and trauma diagnosed on PMCT and TIA, with only minor discrepancies identified. Whilst none of these were critical to providing the medical cause of death, i.e. hanging, arguably some may offer additional information potentially relevant to a coronial investigation, e.g. potential behavioural effects associated with hypothyroidism or old frontal cortex contusions. However, it is often not possible to confirm or quantify the effect such pathologies may have had on an individual from the autopsy findings.

Finally, in a small number of cases, additional information was provided by PMCT, e.g. suspected tablet residue within the stomach in 12% of cases. Such information would assist the pathologist and/or coroner in a decision to order toxicological analyses.

## Summary

Our study provides quantitative data to assist with the ‘real world’ day-to-day decisions being made by death investigation professionals in a common case type. The research has shown that PMCT can augment, to a degree, the postmortem examination in cases of suspected suicidal hanging, primarily by providing an archive of the body and, if present, the ligature. However, the technique should not be used as a replacement for a ‘naked-eye’ external examination by an experienced autopsy pathologist. Furthermore, the research demonstrated that PMCT missed a considerable number of laryngeal fractures. Whilst this may be improved with additional PMCT protocols, radiological methods and/or optimised positioning of the body, all of which could form future research, it would seem reasonable to suggest that standard PMCT examinations should not be completed in isolation to diagnose or exclude neck trauma, not only in hanging cases but across all case types.

## Data Availability

The datasets generated during the current study are not publicly available as the information is the legal property of the Coroner for Northern Ireland.
